# DHX38 restricts chemoresistance by regulating the alternative pre-mRNA splicing of RELL2 in pancreatic ductal adenocarcinoma

**DOI:** 10.1371/journal.pgen.1010847

**Published:** 2023-07-28

**Authors:** Zeru Li, Cheng Qin, Bangbo Zhao, Yuanyang Wang, Tianyu Li, Yutong Zhao, Weibin Wang

**Affiliations:** 1 Department of General Surgery, Peking Union Medical College Hospital, Peking Union Medical College, Chinese Academy of Medical Sciences, Beijing, P.R. China; 2 Key Laboratory of Research in Pancreatic Tumor, Chinese Academy of Medical Sciences, Beijing, P.R. China; Brigham and Women’s Hospital, UNITED STATES

## Abstract

Intron retention plays an important role in cancer progression and chemotherapy resistance and seems to be essential for the maintenance of genome stability in cancer. Here, our goal was to analyze the role of receptor expressed in lymphoid tissue (Relt)-like 2 (RELL2) intron 4 retention in promoting pancreatic ductal adenocarcinoma (PDAC) progression. Our results showed that intron retention (IR) occurs at the fourth intron of RELL2 transcript in gemcitabine resistant PDAC cells, however, the regulatory mechanism and the clinical implications of IR of RELL2 are unclear. Firstly, we found that RELL2 plays an anti-oncogenic role in PDAC by performing in vitro functional assays including cell proliferation, GEM cytotoxicity assay and apoptosis. Subsequently, we identified the upstream gene of RELL2, DEAH-Box Helicase 38 (DHX38), and demonstrated the direct interaction between DHX38 and RELL2 by RIP-qPCR. We also found that altered expression of DHX38 resulted in corresponding changes in intron 4 retention of RELL2. Importantly, we unveiled that overexpression of DHX38 on the basis of knocking down of the fourth intron of RELL2 resulted in an impaired intron 4 intention. Overall, our study identified a new IR site in PDAC, which could be a possible target for PDAC therapy.

## Author summary

Intron retention is a form of alternative splicing which has attracted much attention in recent years. More and more studies have shown that intron retention exists widely and affects 80% of the encoded genes, thus regulating gene expression and physiological and pathological processes. Here, we described the role of alternative splicing gene RELL2 and its upstream splicer DHX38 in pancreatic ductal adenocarcinoma. We found that the expression of DHX38 in pancreatic ductal adenocarcinoma was reduced, which led to the retention of the fourth intron of RELL2 and the occurrence of nonsense-mediated mRNA decay, and ultimately inhibit the apoptosis of pancreatic cancer cells, and promote the occurrence of drug resistance in pancreatic cancer. Our work found a new intron retention gene in pancreatic ductal adenocarcinoma, which provide a probable therapeutic target of pancreatic ductal adenocarcinoma.

## 1. Introduction

Pancreatic ductal adenocarcinoma (PDAC) is currently the fourth leading cause of cancer related death in the USA, with an increasing incidence and poor outcome and constitutes one of the most lethal of the common malignancies with a poor five-year survival rate below 11% [1]. Despite advances in treatment strategies (including surgery, chemotherapy, radiotherapy and targeted therapies) in recent years, the survival rate of PDAC patients still remains a Gordian knot. It is widely known that these four genes are frequently mutated in PDACs: KRAS (94% of tumors), TP53 (64%), SMAD4 (21%) and CDKN2A (17%). Despite these common oncogenic drivers, PDACs still exhibit a high degree of inter-tumor heterogeneity at genomic, metabolic, transcriptomic and histopathological levels. Therefore, it is urgent to explore the underlying mechanisms of PDAC progression.

In addition to somatic DNA mutations, aberrant RNA transcripts can be a major source of genomic disruption in tumors. Intron retention (IR) is a form of alternative splicing (AS) that has been very much studied in non-mammalian species such as plants, fungi, insects and viruses, but has long been neglected in mammalian systems. IR introduces premature stop codons, leading to nonsense-mediated decay and disruption of protein expression. It is commonly believed that mis-splicing leading to the retention of introns has no physiological consequences other than a reduction in gene expression through nonsense-mediated decay. Recent landmark discoveries highlight the critical role of IR in normal and disease-related human biology including cancer. Analyses of exon-specific microarray data from 28 PDAC and 6 normal pancreas tissues, exon skipping (14.3%), alternative first exon use (14%), and intron retention (8.4%) were found as the three most prevalent categories of AS events in PDAC samples [2]. Hilary A. Coller and colleagues unsupervised clustered 76 PDAC patients based on IR events and found two clusters of tumors with significantly different clinical outcomes. However, they draw a different conclusion with previous research, patients with higher levels of intron removal were most likely to have aggressive disease [3,4].

Given that drug resistance in PDAC is a bottleneck in clinical management, we performed RNA-seq in PDAC drug-resistant cell lines and parental cell lines. The presence of IR in receptor expressed in lymphoid tissue (Relt)-like 2 (RELL2) in resistant cell lines was clarified. Previous studies have shown that RELL2 inhibits tumor progression by promoting apoptosis. In our study, firstly, we demonstrated the tumor suppressive effect of RELL2 in PDAC through in vitro assays including cell proliferation, cytotoxicity assays and apoptosis. Subsequently, we confirmed the presence of RELL2 intron 4 retention in PDAC cells and found that IR on RELL2 is directly regulated by (DEAH-Box Helicase 38) DHX38. Based on above consideration, we hope modulating RELL2 splicing will be an potential choice to improve the therapeutic strategies of PDAC patients.

## 2. Results

### 2.1 Identification and Detection of RELL2 Intron Retention in PDAC

**Fig 1:**
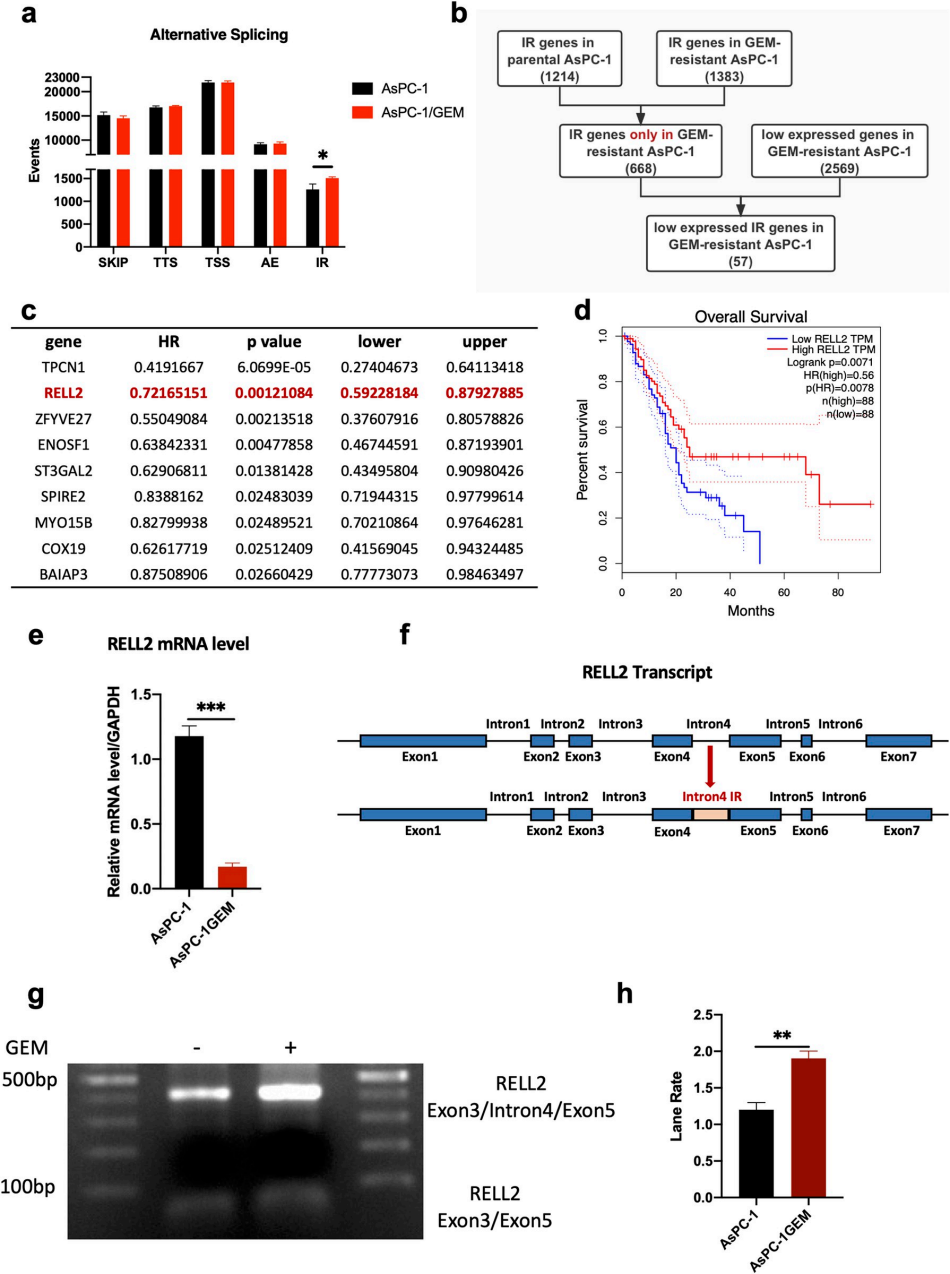
Identification and Detection of RELL2 Intron Retention in PDAC. (**a**) AS events in parental AsPC-1 and GEM-resistant AsPC-1. *, *p < 0*.*05*. (**b**) Screening for 57 IR-related genes that are lowly expressed in GEM-resistant cells. (**c**) Genes of clinical significance in TCGA analysis. (**d**) RELL2 was associated with poor prognosis in PDAC. (**e**) RELL2 was lowly expressed in GEM-resistant AsPC-1. ***, *p < 0*.*001*. (**f**) The IR region of RELL2. (**g, h**) RELL2 undergoes fourth intron retention in GEM-resistant AsPC-1 cells. **, *p < 0*.*01*.

Based on the RNA-seq data, we compared the alternative splicing events occurring in PDAC resistant cells and parental cells. A significant elevation in IR was observed in drug-resistant cells (Fig 1A). Considering that most IRs go through nonsense-mediated decay (NMD), we then analyzed the genes that were lowly expressed in gemcitabine (GEM)-resistant cells and IR-related genes, and obtained 57 IR-related genes that were lowly expressed in GEM-resistant cells (Fig 1B) [5]. TCGA prognostic analysis of these 57 genes revealed that RELL2 was associated with poor prognosis in PDAC, which is consistent with the anti-oncogenic function of RELL2 reported in previous studies (Fig 1C–1E) [6]. Based on above analysis, we screened the regions in AsPC-1 and GEM-resistant AsPC-1 where RELL2 undergoes IR, and found that RELL2 undergoes fourth intron retention in GEM-resistant AsPC-1 cells (Fig 1F–1H). In order to demonstrate that RELL2 mRNA was down-regulated by nonsense mediated decay (NMD) after intron retention occurred, we added NMDI14, a commonly used NMD inhibitor. The results shown that inhibition of NMD led to up-regulation of RELL2 exon3/exon5, and down-regulation of the exon3/intron4/exon5 region (S2 Fig).

### 2.2 RELL2 suppresses proliferation, chemoresistance and promotes apoptosis in PDAC

**Fig 2:**
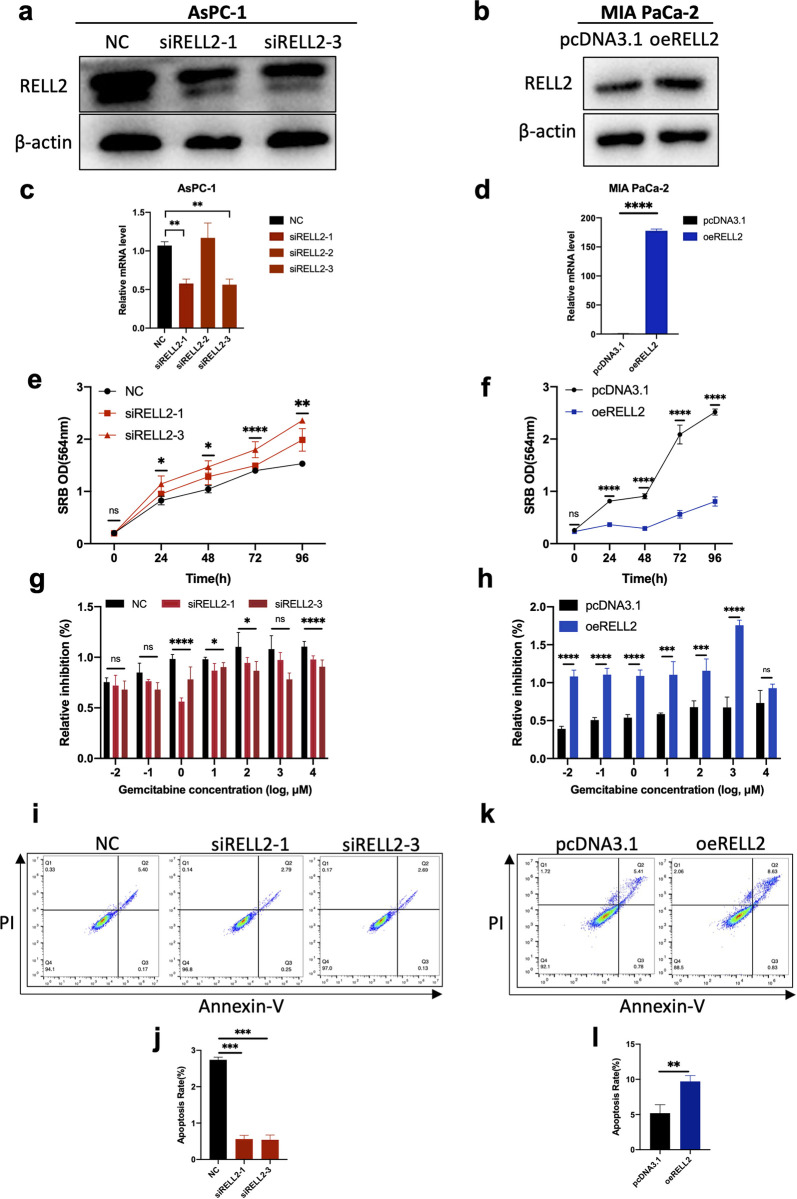
RELL2 suppresses proliferation, chemoresistance and promotes apoptosis in PDAC. (**a**) Transfection efficiency of siRNAs targeting RELL2 in AsPC-1 cells was verified by western blot. (**b**) Transfection efficiency of plasmid targeting RELL2 in MIA PaCa-2 cells was verified by western blot. (**c**) Transfection efficiency of siRNAs targeting RELL2 in AsPC-1 cells was verified by qRT-PCR. **, *p < 0*.*01*. (**d**) Transfection efficiency of plasmid targeting RELL2 in MIA PaCa-2 cells was verified by qRT-PCR. ****, *p < 0*.*0001*. (**e, f**) Proliferation curves of AsPC-1 and MIA Paca-2 transfected with siRELL2 or plasmid for 96 hours using SRB assay. *, *p < 0*.*05*, **, *p < 0*.*01*, ****, *p < 0*.*0001*. (**g, h**) GEM inhibiting rate of AsPC-1 and MIA Paca-2 transfected with siRELL2 or plasmid. *, *p < 0*.*05*, ****, *p < 0*.*0001*. (**i-l**) Cell apoptosis rate of AsPC-1 and MIA Paca-2 transfected with siRELL2 or plasmid. **, *p < 0*.*01*, ***, *p < 0*.*001*.

Next, we performed functional experiments to verify the effects of RELL2 on the malignant biological behavior of PDAC cells. Using qRT-PCR, we obtained the expression profile of RELL2 in 7 commonly used PDAC cell lines at the RNA level and found that RELL2 was relatively high in AsPC-1 expression, relatively low expression in MIA PaCa-2 (S1 Fig). Overexpression plasmid and siRNAs targeting RELL2 were transfected into these two PDAC cell lines and the transfection efficiency was confirmed by qRT-PCR and western blot assays (Fig 2A–2D). Subsequently, we performed *in vitro* functional tests. The results of proliferation experiments showed that knockdown of RELL2 could significantly promote the *in vitro* proliferation of AsPC-1 (Fig 2E), overexpression of RELL2 can inhibit the in vitro proliferation ability of MIA PaCa-2 (Fig 2F). GEM cytotoxicity assay showed that knockdown of RELL2 could inhibit the sensitivity of AsPC-1 to GEM, while overexpression of RELL2 could induce the sensitivity of PDAC cells to GEM (Fig 2G and 2H). Apoptosis experiments showed that knockdown of RELL2 could significantly inhibit the apoptosis rate of PDAC cells (Fig 2I and 2J), and overexpression of RELL2 could significantly promote the apoptosis ability of PDAC cells (Fig 2K and 2L). Together, our data revealed that RELL2 could suppress the PDAC cells proliferation and disturbed drug resistance.

### 2.3 DHX38 directly regulates RELL2 in PDAC cells

**Fig 3:**
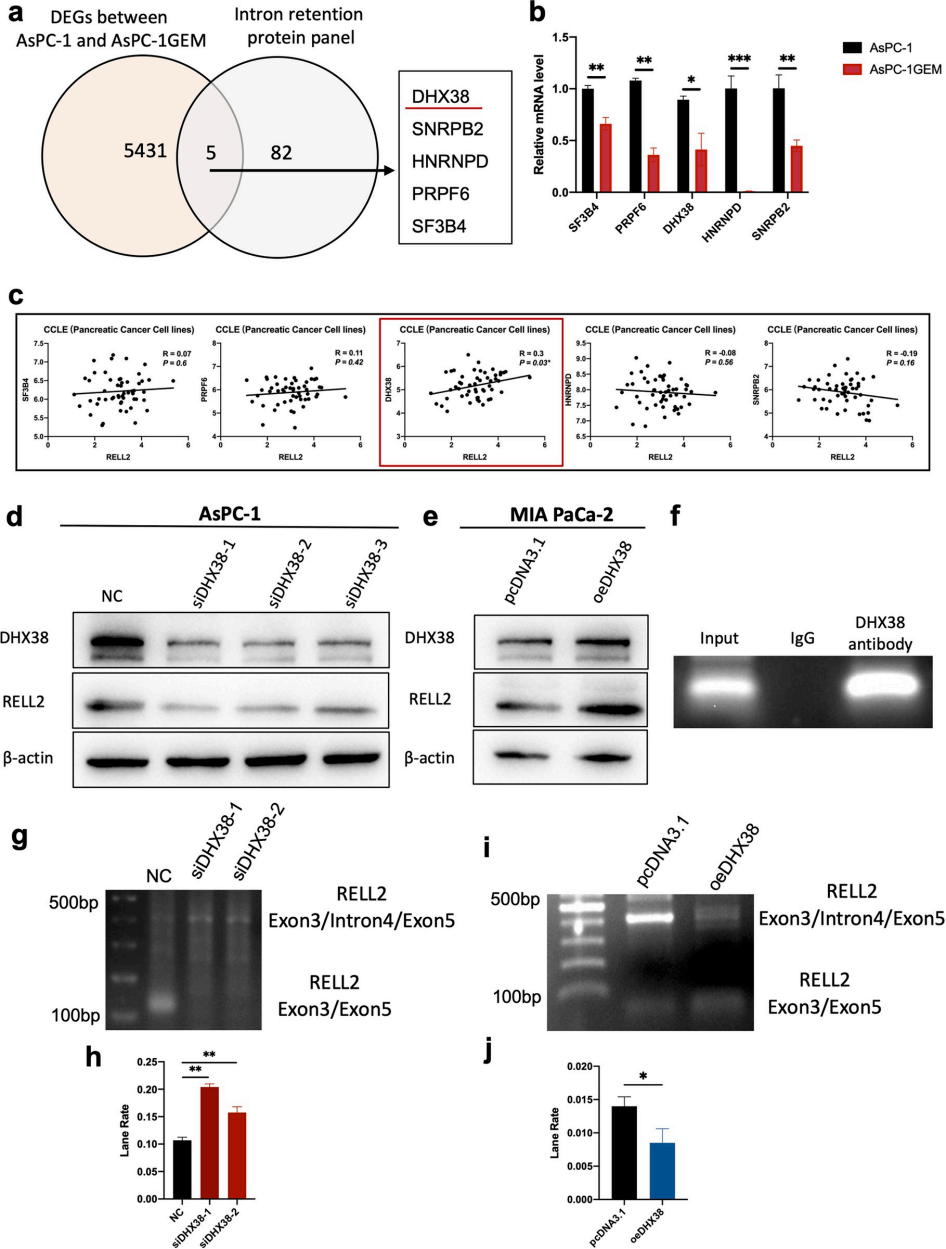
DHX38 directly regulates RELL2 in PDAC cells. (**a**) Screening for DHX38 in DEGs (AsPC-1 and Gem-resistant AsPC-1) and IR protein panel. (**b**) Expression level of DHX38, SF3B4, PRPF6, HNRNPD, SNRPB2 in parental AsPC-1 and GEM-resistant AsPC-1. *, *p < 0*.*05*, **, *p < 0*.*01*, ***, *p < 0*.*001*. (**c**) The correlation of the five genes with RELL2 in PDAC cells. (**d**) Transfection efficiency of siRNAs targeting DHX38 in AsPC-1 cells and expression of RELL2 were verified by western blot. (**e**) Transfection efficiency of plasmid targeting DHX38 in MIA PaCa-2 cells and expression of RELL2 were verified by western blot. (**f**) The direct correlation of RELL2 and DHX38 was verified by RIP assay. (**g-j**) The regulation of DHX38 on RELL2 intron 4 retention. ***, *p < 0*.*001*.

After the preliminary exploration of the key role of RELL2 was completed, we turned to further explore the upstream gene that regulate the expression and IR progress of RELL2. We analyzed the differentially expressed genes (DEGs) between parental AsPC-1 and GEM-resistant AsPC-1 and found that five of these genes were closely associated with IR event (Fig 3A and 3B). Further analysis of the correlation of the above five genes with RELL2 in PDAC cell lines revealed that DHX38 was positively correlated with RELL2 (Fig 3C). The relationship between DHX38 and RELL2 was verified by western-blot. The results showed that knockdown of DHX38 would decrease the expression of RELL2, while overexpression of DHX38 leads to RELL2 induced (Fig 3D and 3E). Further, we performed RNA co-immunoprecipitation-qPCR (RIP-qPCR) to verify that DHX38 can bind to the fourth intron region of the RELL2 mRNA, and found that compared with IgG, the DHX38 antibody group could significantly bind to the fourth intron region of RELL2 pre-mRNA (Fig 3F). After clarifying the regulatory role of DHX38 on RELL2, we further verified the effect of DHX38 on IR event of RELL2. Knocking down of DHX38 increased RELL2 intron 4 retention, and conversely overexpression of DHX38 decreased RELL2 intron 4 retention (Fig 3G–3J). Overall, the above observation suggested that DHX38 directly controlled the expression level and intron 4 retention of RELL2.

### 2.4 DHX38 inhibits PDAC progression by regulating RELL2 pre-mRNA

**Fig 4:**
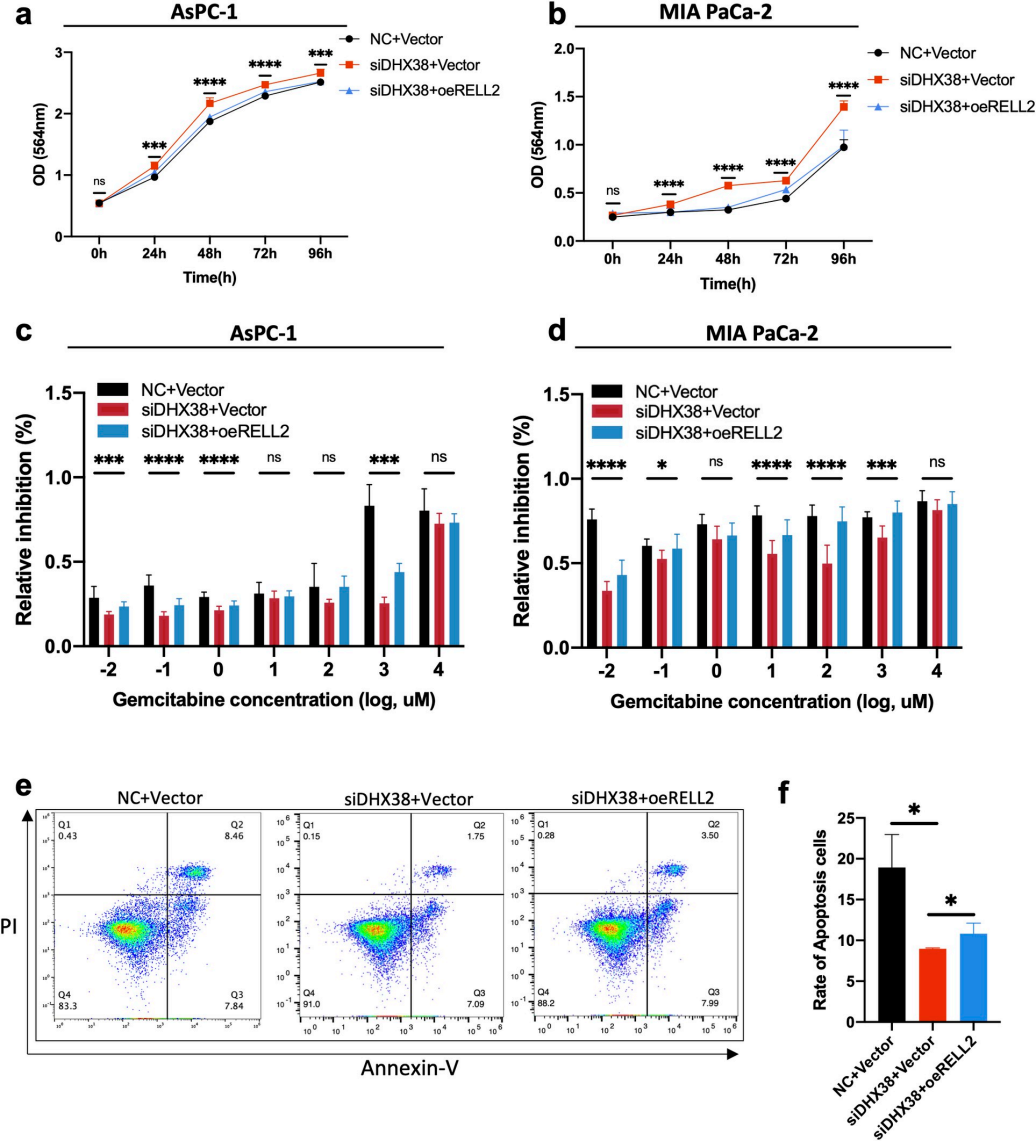
DHX38 inhibits PDAC progression by regulating RELL2 pre-mRNA. (**a, b**) The effects of RELL2 overexpression on cell proliferation in AsPC-1 and MIA PaCa-2 cells with siRNA targeting DHX38 transfected. ***, *p < 0*.*001*, ****, *p < 0*.*0001*. (**c, d**) The effects of RELL2 overexpression on GEM inhibitory rate in AsPC-1 and MIA PaCa-2 cells with siRNA targeting DHX38 transfected. *, *p < 0*.*05*, ***, *p < 0*.*001*, ****, *p < 0*.*0001*. (**e, f**) The effects of RELL2 overexpression on cell apoptosis rate in AsPC-1 cells with siRNA targeting DHX38 transfected. *, *p < 0*.*05*.

In previous studies, DHX38 was found to promote apoptosis and play an inhibitory role in tumors [7]. We therefore hypothesize that DHX38 inhibits PDAC progression via RELL2. After knocking down DHX38 in AsPC-1 and MIA PaCa-2 cells, cell proliferation was increased, followed by overexpression of RELL2, and cell proliferation was partially reduced (Fig 4A and 4B). GEM cytotoxicity assay revealed that knockdown of DHX38 could suppress the sensitivity of these two cell lines to GEM, while overexpression of RELL2 could partly induce the sensitivity of PDAC cells to GEM (Fig 4C and 4D). The relevant role of DHX38 and RELL2 in PDAC was also verified by flow cytometry. The cell apoptosis rate was decreased after knocking down DHX38, and this phenomenon would be rescued by overexpression of RELL2 (Fig 4E and 4F). Together, these findings supported the notion that DHX38 suppresses PDAC progression via regulating RELL2.

### 2.5 DHX38 promotes RELL2 intron 4 retention in PDAC cells

**Fig 5:**
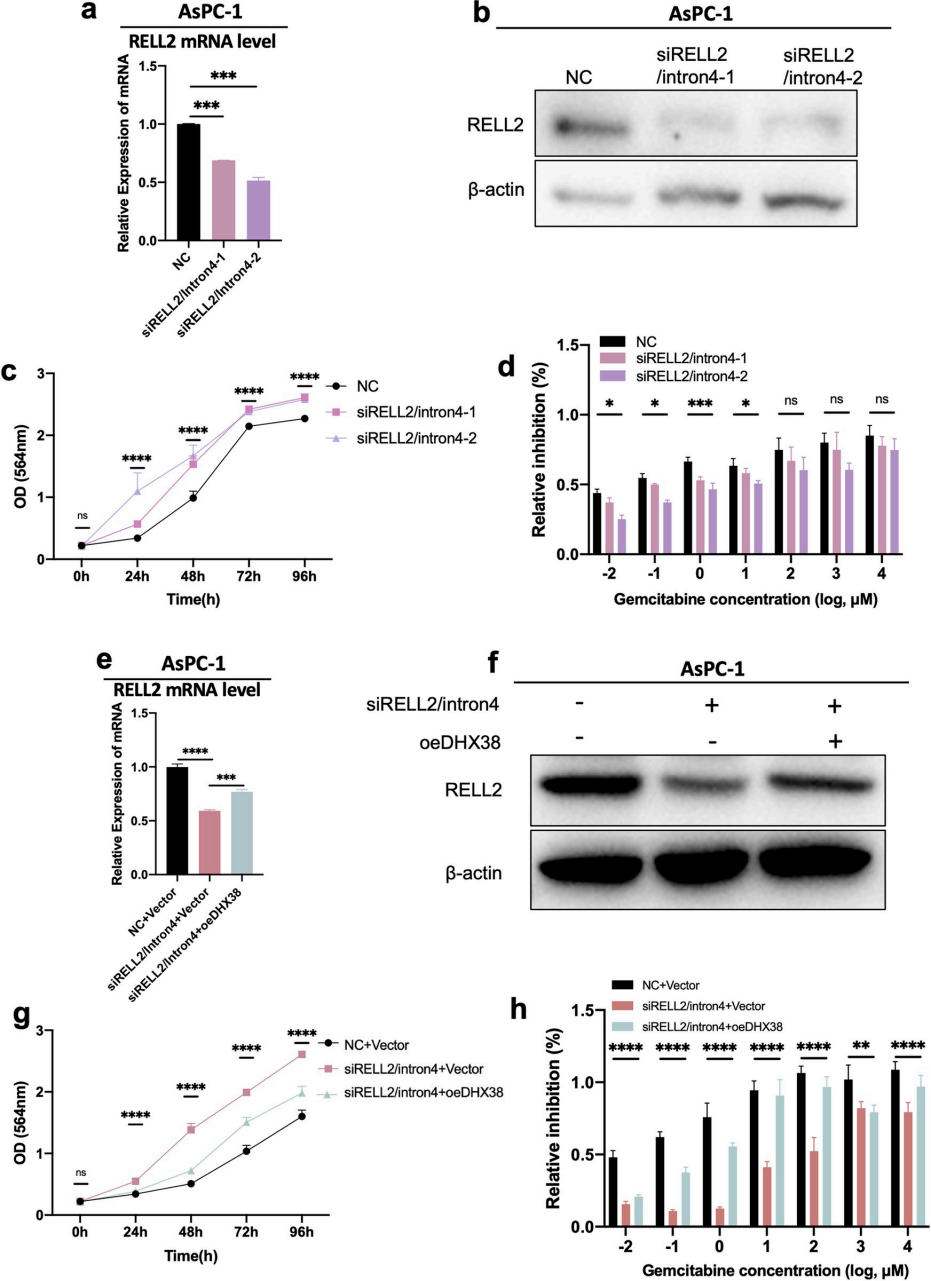
DHX38 promotes RELL2 intron 4 retention in PDAC cells. (**a**) The effect of siRNAs targeting RELL2 intron 4 on mRNA level of RELL2. ***, *p < 0*.*001*. (**b**) The effect of siRNAs targeting RELL2 intron 4 on protein level of RELL2. (**c**) Proliferation curves of AsPC-1 transfected with siRELL2-intron 4 for 96 hours using SRB assay. ****, *p < 0*.*0001*. (**d**) GEM inhibitory rate of AsPC-1 transfected with siRELL2-intron 4. *, *p < 0*.*05*, ***, *p < 0*.*001*. (**e**) The effects of DHX38 overexpression on mRNA level of RELL2 in AsPC-1 with siRNA targeting RELL2 intron 4 transfected. ***, *p < 0*.*001*. (**f**) The effects of DHX38 overexpression on protein level of RELL2 in AsPC-1 with siRNA targeting RELL2 intron 4 transfected. (**g**) The effects of DHX38 overexpression on cell proliferation in AsPC-1 siRNA targeting RELL2 intron 4 transfected. ****, *p < 0*.*0001*. (**h**) The effects of DHX38 overexpression on GEM-inhibitory rate in AsPC-1 siRNA targeting RELL2 intron 4 transfected. **, *p < 0*.*01*, ****, *p < 0*.*0001*.

In the above study, we demonstrated the function of RELL2 intron 4 retention in PDAC cells and the regulation of DHX38 on RELL2 expression and function. Next, we focused on the relationship between DHX38 and the fourth intron of RELL2. Firstly, we designed siRNAs targeting the fourth intron of RELL2 and verified by western-blot and qPCR that knocking down the fourth intron of RELL2 decreased the expression level of RELL2 (Fig 5A and 5B). Secondly, *in vitro* functional assays showed that knockdown of the fourth intron of RELL2 increased the proliferation level and decreased the inhibition rate of gemcitabine of PDAC cells (Fig 5C and 5D). We then overexpressed DHX38 on the basis of knocking down the fourth intron of RELL2 to restrict intron 4 retention event. The results showed that overexpression of DHX38 would rescue the expression of RELL2 on both protein level and mRNA level (Fig 5E and 5F) Proliferation assays showed that overexpression of DHX38 on the basis of knocking down the fourth intron of RELL2 led to a decrease in cell proliferation, meanwhile, cytotoxicity assays showed that overexpression of DHX38 on the basis of knocking down the fourth intron of RELL2 made PDAC cells more sensitive to GEM (Fig 5G and 5H).

**Fig 6:**
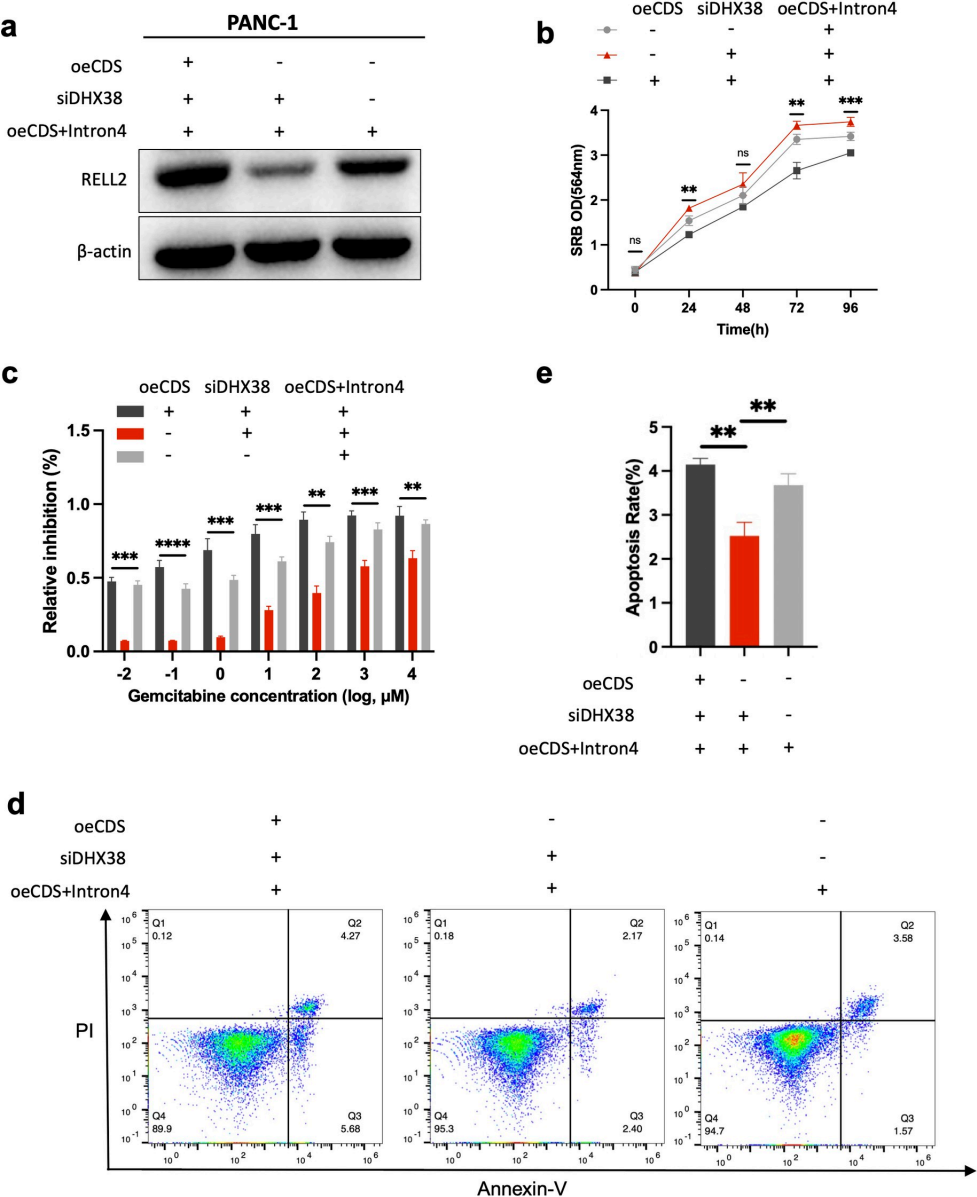
RELL2 intron retention effects cellular phenotypes directly. (**a**) The effect of siDHX38, overexpression of CDS region or CDS+intron4 region on protein level of RELL2. (**b**) Proliferation curves of PANC-1 after knocking down DHX38, overexpression of CDS region or CDS+intron4 region for 96 hours using SRB assay. **, *p < 0*.*01*,***, *p < 0*.*001*. (**c**) GEM inhibitory rate of PANC-1 after knocking down DHX38, overexpression of CDS region or CDS+intron4 region. **, *p < 0*.*01*, ***, *p < 0*.*001*. ****, *p < 0*.*0001*. (**d, e**) Cell apoptosis rate of PANC-1 after knocking down DHX38, overexpression of CDS region or CDS+intron4 region. **, *p < 0*.*01*.

To provide more direct evidence for the role of RELL2 intron retention on cellular phenotype, we selected the cell line with the lowest RELL2 expression, PANC-1, and overexpressed the CDS+intron 4 region of RELL2. On this basis, we knocked down DHX38, and observed that the expression of RELL2 was decreased due to the deletion of splicing effect of DHX38. Subsequently, we overexpressed the CDS region of RELL2, the expression of RELL2 was rescues obviously (Fig 6A). In addition, we performed cellular phenotypes including proliferation, cytotoxicity assay and cell apoptosis, the results were consistent (Fig 6B–6E).

Altogether, we demonstrate that RELL2 undergoes intron 4 retention in PDAC GEM-resistant cells, a process that is directly regulated by DHX38.

## 3. Discussion

The concept of alternative splicing has progressively become one of the hot spots in human gene regulation research since the discovery of mRNA splicing by Nobel Prize winners over 40 years ago [8,9]. High-throughput sequencing technologies have shown that 92%-95% of multi-exon genes in human genome undergo AS, resulting in multiple heterodimers. AS is often abnormally altered in disease states, especially tumors [10,11]. Tumor cells exhibit significant splicing alterations, including IR. For a long time, intron retention in tumors was thought to be merely an artefact of dysfunctional spliceosomes. Technological advances over the last decade have provided the opportunity to explore the role of IR in tumor cells [3,12]. Numerous studies have revealed dysregulated cellular mechanisms leading to paradoxical and pathological IR, which is not just a mechanism of gene regulation but can mediate the pathogenesis of cancer and therapeutic resistance in a variety of human diseases. IR means a mature mRNA transcript that retains at least one intron-in a sense holding on to its ‘junk bond (s)’, because introns once been referred to as ‘junk DNA’. It has been shown that IR affect approximately 80% of human protein coding genes [13]. For example, IR is tissue and cell-type specific. It occurs frequently in neural and immune cell types, but is less observed in embryonic stem cells and muscle [14]. In recent years, the role of IR has also been highlighted in cancer research. Zinc finger CCCH-type, RNA binding motif and serine/arginine rich 2 (ZRSR2) loss leads to impaired minor intron excision of leucine zipper-like transcription regulator 1 (LZTR1), and enhances hematopoietic stem cell self-renewal [15,16]. One of the mechanisms of solid tumors react to hypoxia is impede energy-consuming processes including alternative splicing and translation. In head and neck cancer, reduction of core splicing factor expression caused by hypoxia (i.e., SF1, SRSF1, SRSF3, and SRSF7) results in nearly 90% of IR-affected genes displaying high retention of introns in hypoxic compared with normal cells [17]. Targeted therapeutic options for intron retention sites have been partially investigated in previous studies. Promoter DNA methylation silences exon inclusion-regulator CUGBP Elav-like family member 2 (CELF2) expression in pancreatic, gastric and breast cancer cells, and epigenetic silencing of CELF2 is associated with poor patient prognosis. 5’-aza-2’ deoxycytidine restores the IR caused by CELF2 and inhibits tumor cell growth [18]. Non-POU domain-containing octamer-binding protein (NONO), a Drosophila behavior human splicing (DBHS) protein, was found highly expressed in glioblastoma (GBM). The loss of NONO altered the splicing of GPX1 and led to inhibition of proliferation and invasion of GBM. The small molecule inhibitor Auranofin blocked NONO activity and inhibited GBM tumor growth in an in vivo orthotopic xenograft model [19].

RELL2 is originally identified as a homologue of RELT, a member of tumor necrosis factor receptor superfamily that is selectively expressed in hematological tissue. RELL2 is localized in the plasma membrane and the expression of RELL2 in human tissues such as brain, testis, spleen, thymus, leukocytes and placenta has been demonstrated [20]. It has been found that targeted regulation of RELL2 by microRNA-18a is implicated in the anti-metastatic effect of polyphyllin VI in breast cancer cells [6]. However, very little is known about the function of RELL2 in PDAC.

In our study, we demonstrated that intron retention events were significantly higher in drug resistant PDAC cells than in parental cells, and RELL2 associates with good prognosis of PDAC patients. We analyzed the six intron regions of RELL2 and ultimately found that intron retention events of RELL2 in PDAC occurred at the fourth intron region. Through in vitro functional assays, we found that RELL2 plays an anti-oncogenic role in PDAC. Further, we identified the upstream gene of RELL2, DHX38, by analyzing the DEGs between GEM-resistant cells and parental cells and intron retention protein panel. Subsequently, we demonstrated the direct interaction between DHX38 and RELL2 by RIP-PCR, and found that altered expression of DHX38 resulted in corresponding changes in intron 4 retention of RELL2. Importantly, we found that overexpression of DHX38 promoting normal splicing of RELL2 pre-mRNA and RELL2 protein synthesis.

Previous studies have also demonstrated the anti-tumor role of RELL2 and we got a consistent conclusion through our series of exploration [21]. The novelty of our study is the first exploration of the occurrence of intron retention events of RELL2 in PDAC, and the demonstration of a direct interaction between RELL2 and the protein splicing molecule DHX38, which has not been previously reported. Overall, our study has identified a new intron retention site in PDAC, which could be a possible target for PDAC therapy.

## 4. Materials and methods

### 4.1 Cell lines and cell culture

Seven common PDAC cell lines and a pancreatic ductal epithelial cell line were used in this article. All the cell lines were purchased from American Type Culture Collection (ATCC). SW1990 was cultured in RPMI-1640 medium (Hyclone, Utah, Logan, USA). MIA PaCa-2, AsPC-1, HPNE, PANC-1, and T3M4 were cultured in DMEM/high glucose (Hyclone, Utah, Logan, USA). Capan-1 and CFPAC were cultured in IMDM (Hyclone, Utah, Logan, USA). The culture medium of Capan-1 was added with 20% fetal bovine serum and others were supplemented with 10% fetal bovine serum.

### 4.2 siRNA and plasmid transfection

Gene-specific siRNAs and nonsense control were provided by Tsingke (Beijing, China). PDAC cells were transfected with siRNA using Lipofectamine 8000 (Invitrogen, California, USA). After the knockdown efficiency was confirmed by quantitative RT-PCR (qRT-PCR) and western blot, the cells were used for subsequent experiment. The overexpression plasmids were provided by RiboBio (Guangzhou, China). The plasmids were transfected into the cells with Lipofectamine 8000 as recommended by the manufacturer. The sequences of primers and siRNAs used were listed in S1 Table.

### 4.3 *In vitro* cell proliferation assay

Cells after treatments were seeded in 96-well plates and assessed using sulforhodamine B (SRB) (Sigma, St. Louis, USA) assay under the time gradient [22]. The cultured cells were fixed with 10% trichloroacetic acid for 20min and stained with 0.4% SRB for 30min at room temperature. Cells were then repeatedly washed with 1% acetic acid and the dye was dissolved in 10mM Tris. OD value at 564 nm was measured at last.

### 4.4 *In vitro* cell cytotoxicity assay

Cells were seeded into 96-well plates for 8 hours before adding GEM. The concentration gradient of GEM were 0, 1nM, 10nM, 100nM, 1μM, 10μM, 100μM and 1M. After treating for 48 hours, drug-containing culture medium was replaced by fresh medium which contained 10% CCK-8 (Dojindo, Japan). Place the plates in incubator under 37°C for 2 hours and determine its light absorption value at 450nm and 630nm using an enzyme-linked immunosorbent detector (Invitrogen, Thermo Fisher Scientific, USA). The difference between absorbance values at 450nm and 630nm indirectly reflects the number of living cells.

### 4.5 Apoptosis analysis

Cell apoptosis was tested by using Annexin-V/PE Apoptosis Kit (YISHAN Biotechnology Co., LTD, Shanghai, China). Briefly, cells were plated into a 6-well plate for 24h. After treated with siRNA or plasmid for 48h, cells were collected and washed with PBS for 3 times, and then resuspended by annexin V-binding buffer and 1mg/ml PI and Annexin-V. After incubation for 10 min in the dark, the cells were analyzed by flow cytometry (Attune Nxt 2L-BR, Thermo, USA) with 1h.

### 4.6 Quantitative real-time polymerase chain assay

Total RNA was extracted by TRIzol regent (Invitrogen, CA, USA) and processed for reverse transcription and quantitative PCR using a Reverse Transcription System (Promega, Madison, WI, USA) and a One Step PrimeScripttm RT-PCR Kit (Vazyme, Nanjing, China) according to the manufacturer’s instructions. The 2^-ΔCt^ method was used to quantify fold changes with normalization to *ACTB*. Detailed information on the primer sequences is shown in S2 Table.

### 4.7 Western blot assay

Whole cell lysates were obtained with RIPA lysis buffer (Applygen, Beijing, China) containing 1% protease and phosphatase inhibitors (Sigma-Aldrich, St. Louis, USA) on ice. The cell lysates were centrifuged at 12, 000 rpm for 15 min at 4°C to remove undissolved impurities and collect the supernatants. The protein concentration was quantified using a BCA protein assay kit (Beyotime, Shanghai, China). Then, proteins were separated by 10% SDS-PAGE and transferred to 0.22μm polyvinylidene fluoride (PVDF) membranes. The non-specific binding sites on the membrane were blocked with 5% milk for 1h. After blocking, the membrane was first incubated with the primary antibodies (S3 Table) overnight at 4°C and then with the secondary antibody at room temperature for 1h. Finally, super-sensitive ECL assay kit (Beyotime, Shanghai, China) was used to show the immune response.

### 4.8 cDNA amplification

Endogenous cDNA of RELL2 was amplified by 2xTaq Plus Master Mix II (Vazyme, Jiangsu, China) with primers targeting intron 4 of RELL2. The following PCR conditions were used: Initial denaturation at 95°C for 5 min, followed by 25 cycles consisting of denaturation (95°C for 40 s), annealing (2 min) and extension (72°C for 1 min) and a final extension step at 72°C for 7 min.

### 4.9 DNA agarose gel electrophoresis

The conventional agarose gel electrophoresis analyses were performed essentially as reported [23]. 1% gels were made using agarose (Sigma-Aldrich, St. Louis, USA) and run for 40min-1h at a 120V constant voltage in 1xTBE buffer (Beyotime, Shanghai, China). The gels were stained with Ultra GelRed (Vazyme, Jiangsu, China) and scanned. Images were developed and edited using ImageJ.

### 4.10 RNA immunoprecipitation (RIP)

RNA immunoprecipitation (RIP) experiment was performed using Magna RIP RNA-Binding Protein Immunoprecipitation Kit (Millipore, USA) according to the manufacturer’s instructions. Briefly, PDAC cells were lysed by RIP lysis buffer and then cell lysates were immunoprecipitated with protein A/G magnetic beads conjugated to anti-DHX38 antibody or normal rabbit IgG at 4°C overnight. After RNA purification, fold enrichment of the target region was determined after normalization to the input and compared with the IgG control.

### 4.11 Statistical analysis

The GraphPad Prism version 9.0 (Graphpad, Inc., Chicago, IL) was used for data analysis and graphical representation. Data are presented as the means ± standard deviation. Student’s t test and ANOVA were used for comparisons of significant differences between groups. Survival analyses were performed using the Kaplan-Meier method and assessed using the log-rank test with R version 4.1.2. All *P* values less than 0.05 were considered statistically significant (*, *P*<0.05).

## 5. Conclusions

**Fig 7:**
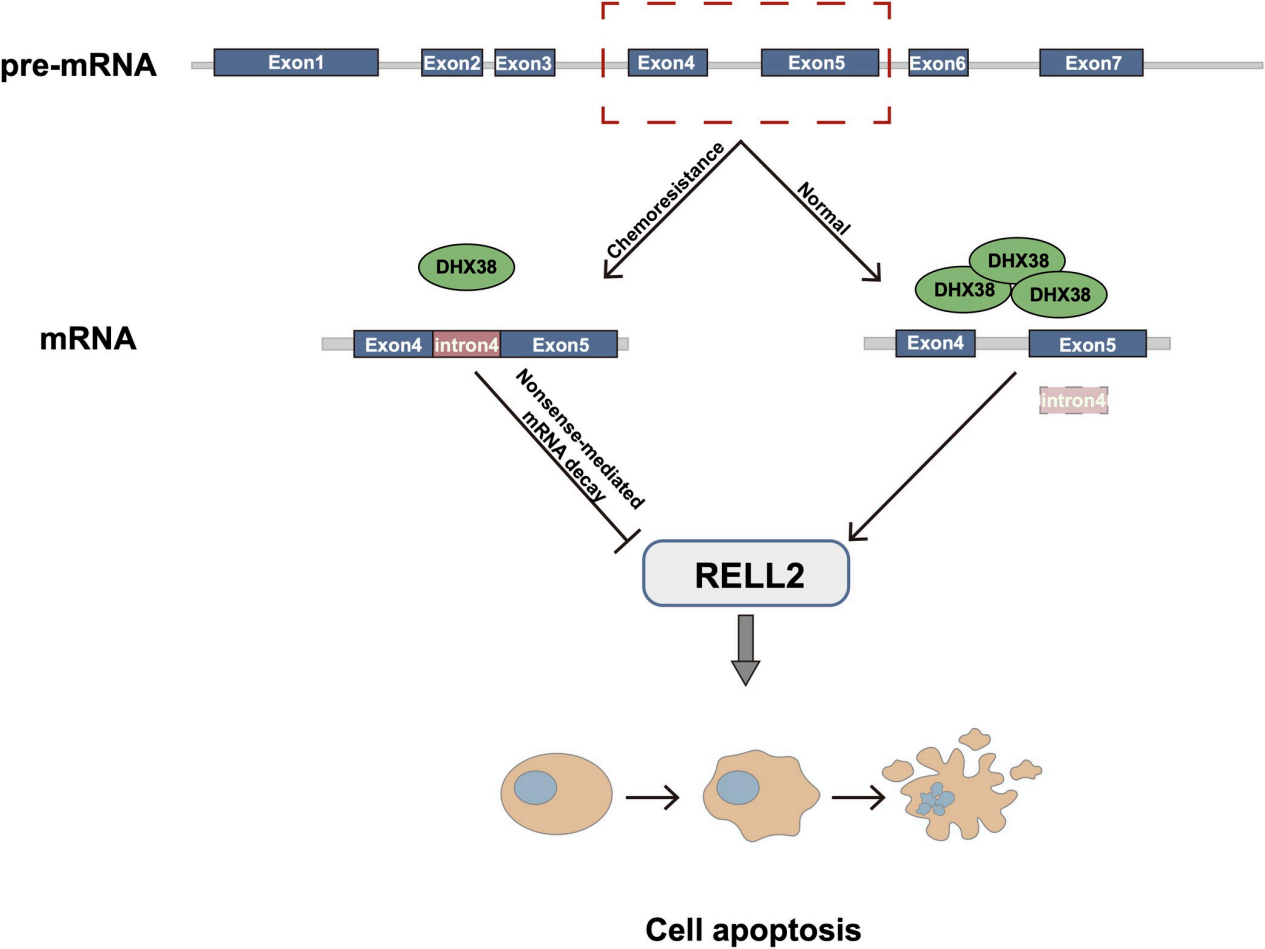
RELL2 undergoes intron 4 retention in PDAC cells and that the occurrence of this event is regulated by the upstream gene DHX38, and leads to dysregulation of the anti-oncogenic function of RELL2, promoting PDAC progression.

In summary, our work identified that RELL2 undergoes intron 4 retention in PDAC cells and that the occurrence of this event is regulated by the upstream gene DHX38, and leads to dysregulation of the anti-oncogenic function of RELL2, promoting PDAC progression (Fig 7).

## Supporting information

S1 Fig The mRNA expression profile of RELL2 in PDAC cell lines. (TIFF)

S2 Fig The effect of nonsense-decay inhibitor NMDI14 on RELL2 intron retention. (TIFF)

S1 Table The sequences of siRNA used in this article. (DOCX)

S2 Table The sequences of primers used in this article. (DOCX)

S3 Table Data of antibodies used in this article. (DOCX)

## References

[1] Siegel RL, Miller KD, Fuchs HE, Jemal A. Cancer Statistics, 2021. CA Cancer J Clin. 2021;71(1):7–33. doi: 10.3322/caac.21654 33433946

[2] Wang J, Dumartin L, Mafficini A, Ulug P, Sangaralingam A, Alamiry NA, et al. Splice variants as novel targets in pancreatic ductal adenocarcinoma. Sci Rep. 2017;7(1):2980. doi: 10.1038/s41598-017-03354-z 28592875 PMC5462735

[3] Dvinge H, Bradley RK. Widespread intron retention diversifies most cancer transcriptomes. Genome Med. 2015;7(1):45. doi: 10.1186/s13073-015-0168-9 26113877 PMC4480902

[4] Tan DJ, Mitra M, Chiu AM, Coller HA. Intron retention is a robust marker of intertumoral heterogeneity in pancreatic ductal adenocarcinoma. NPJ Genom Med. 2020;5(1):55. doi: 10.1038/s41525-020-00159-4 33311498 PMC7733475

[5] Lee G, Zheng Y, Cho S, Jang C, England C, Dempsey JM, et al. Post-transcriptional Regulation of De Novo Lipogenesis by mTORC1-S6K1-SRPK2 Signaling. Cell. 2017;171(7):1545–58.e18. doi: 10.1016/j.cell.2017.10.037 29153836 PMC5920692

[6] Wang P, Yang Q, Du X, Chen Y, Zhang T. Targeted regulation of Rell2 by microRNA-18a is implicated in the anti-metastatic effect of polyphyllin VI in breast cancer cells. Eur J Pharmacol. 2019;851:161–73. doi: 10.1016/j.ejphar.2019.02.041 30817902

[7] Chen X, Lin L, Chen G, Yan H, Li Z, Xiao M, et al. High Levels of DEAH-Box Helicases Relate to Poor Prognosis and Reduction of DHX9 Improves Radiosensitivity of Hepatocellular Carcinoma. Front Oncol. 2022;12:900671. doi: 10.3389/fonc.2022.900671 35814441 PMC9256992

[8] Chow LT, Gelinas RE, Broker TR, Roberts RJ. An amazing sequence arrangement at the 5’ ends of adenovirus 2 messenger RNA. Cell. 1977;12(1):1–8. doi: 10.1016/0092-8674(77)90180-5 902310

[9] Berget SM, Moore C, Sharp PA. Spliced segments at the 5’ terminus of adenovirus 2 late mRNA. Proc Natl Acad Sci U S A. 1977;74(8):3171–5. doi: 10.1073/pnas.74.8.3171 269380 PMC431482

[10] Wang ET, Sandberg R, Luo S, Khrebtukova I, Zhang L, Mayr C, et al. Alternative isoform regulation in human tissue transcriptomes. Nature. 2008;456(7221):470–6. doi: 10.1038/nature07509 18978772 PMC2593745

[11] Barbosa-Morais NL, Irimia M, Pan Q, Xiong HY, Gueroussov S, Lee LJ, et al. The evolutionary landscape of alternative splicing in vertebrate species. Science. 2012;338(6114):1587–93. doi: 10.1126/science.1230612 23258890

[12] Schmitz U, Pinello N, Jia F, Alasmari S, Ritchie W, Keightley MC, et al. Intron retention enhances gene regulatory complexity in vertebrates. Genome Biol. 2017;18(1):216. doi: 10.1186/s13059-017-1339-3 29141666 PMC5688624

[13] Middleton R, Gao D, Thomas A, Singh B, Au A, Wong JJ, et al. IRFinder: assessing the impact of intron retention on mammalian gene expression. Genome Biol. 2017;18(1):51. doi: 10.1186/s13059-017-1184-4 28298237 PMC5353968

[14] Braunschweig U, Barbosa-Morais NL, Pan Q, Nachman EN, Alipanahi B, Gonatopoulos-Pournatzis T, et al. Widespread intron retention in mammals functionally tunes transcriptomes. Genome Res. 2014;24(11):1774–86. doi: 10.1101/gr.177790.114 25258385 PMC4216919

[15] Yoshida K, Sanada M, Shiraishi Y, Nowak D, Nagata Y, Yamamoto R, et al. Frequent pathway mutations of splicing machinery in myelodysplasia. Nature. 2011;478(7367):64–9. doi: 10.1038/nature10496 21909114

[16] Inoue D, Polaski JT, Taylor J, Castel P, Chen S, Kobayashi S, et al. Minor intron retention drives clonal hematopoietic disorders and diverse cancer predisposition. Nat Genet. 2021;53(5):707–18. doi: 10.1038/s41588-021-00828-9 33846634 PMC8177065

[17] Brady LK, Wang H, Radens CM, Bi Y, Radovich M, Maity A, et al. Transcriptome analysis of hypoxic cancer cells uncovers intron retention in EIF2B5 as a mechanism to inhibit translation. PLoS Biol. 2017;15(9):e2002623. doi: 10.1371/journal.pbio.2002623 28961236 PMC5636171

[18] Piqué L, Martinez de Paz A, Piñeyro D, Martínez-Cardús A, Castro de Moura M, Llinàs-Arias P, et al. Epigenetic inactivation of the splicing RNA-binding protein CELF2 in human breast cancer. Oncogene. 2019;38(45):7106–12. doi: 10.1038/s41388-019-0936-x 31409895

[19] Wang X, Han M, Wang S, Sun Y, Zhao W, Xue Z, et al. Targeting the splicing factor NONO inhibits GBM progression through GPX1 intron retention. Theranostics. 2022;12(12):5451–69. doi: 10.7150/thno.72248 35910786 PMC9330516

[20] Cusick JK, Xu LG, Bin LH, Han KJ, Shu HB. Identification of RELT homologues that associate with RELT and are phosphorylated by OSR1. Biochem Biophys Res Commun. 2006;340(2):535–43. doi: 10.1016/j.bbrc.2005.12.033 16389068

[21] Sica GL, Zhu G, Tamada K, Liu D, Ni J, Chen L. RELT, a new member of the tumor necrosis factor receptor superfamily, is selectively expressed in hematopoietic tissues and activates transcription factor NF-kappaB. Blood. 2001;97(9):2702–7. doi: 10.1182/blood.v97.9.2702 11313261

[22] Vichai V, Kirtikara K. Sulforhodamine B colorimetric assay for cytotoxicity screening. Nat Protoc. 2006;1(3):1112–6. doi: 10.1038/nprot.2006.179 17406391

[23] Mukai T, Yoneji T, Yamada K, Fujita H, Nara S, Su’etsugu M. Overcoming the Challenges of Megabase-Sized Plasmid Construction in Escherichia coli. ACS Synth Biol. 2020;9(6):1315–27. doi: 10.1021/acssynbio.0c00008 32459960

