# Supplementary figures and images for: DHX38 restricts chemoresistance by regulating the alternative pre-mRNA splicing of RELL2 in pancreatic ductal adenocarcinoma
Source: PLoS Genet. 2023 Jul 28;19(7):e1010847. doi: 10.1371/journal.pgen.1010847 (PMC10381071; doi:10.1371/journal.pgen.1010847)

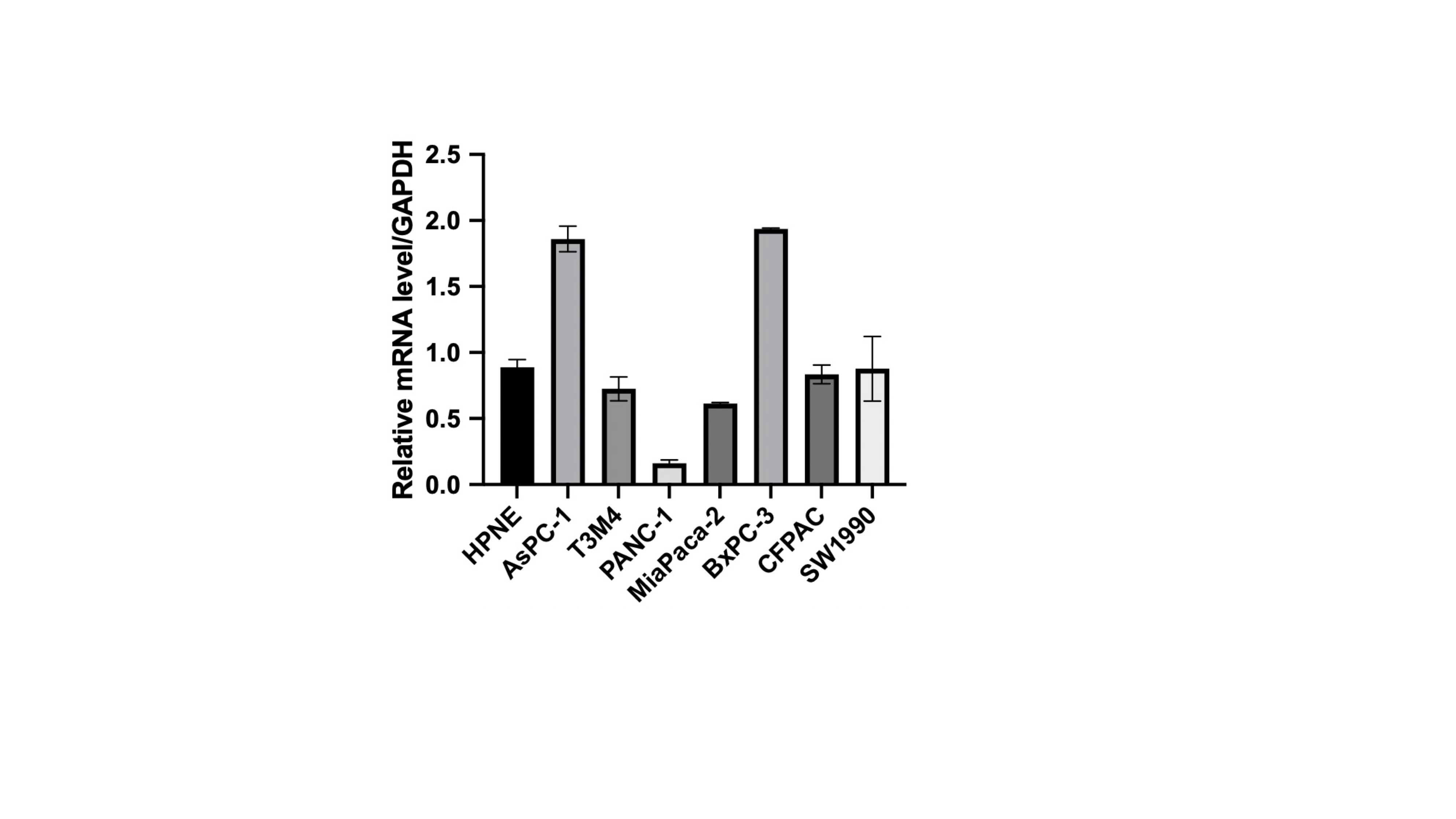

Supplement: S1 Fig — (TIFF) [file pgen.1010847.s001.tiff]

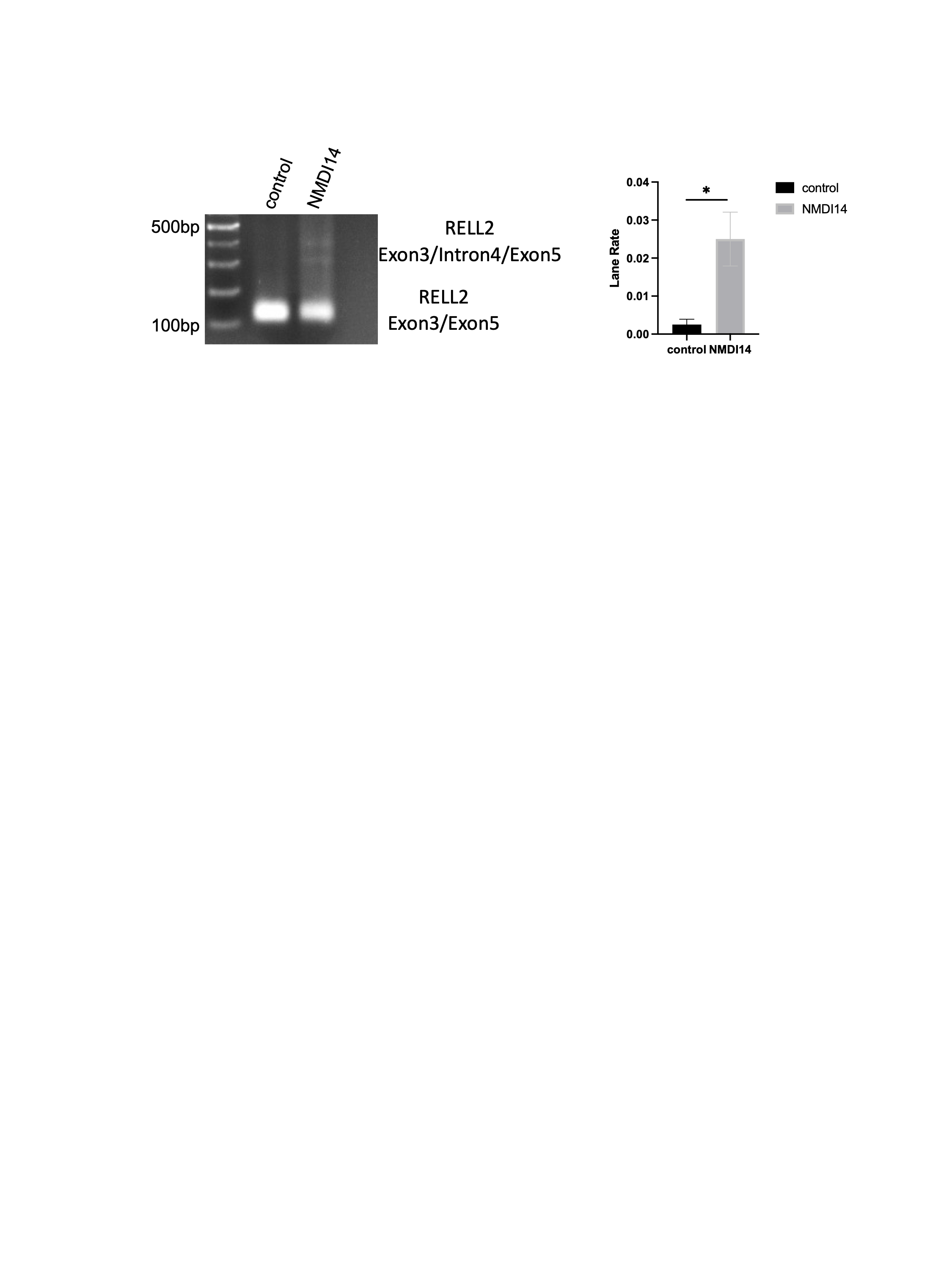

Supplement: S2 Fig — (TIFF) [file pgen.1010847.s002.tiff]
