# Supplementary material for: DHX38 restricts chemoresistance by regulating the alternative pre-mRNA splicing of RELL2 in pancreatic ductal adenocarcinoma
Source: PLoS Genet. 2023 Jul 28;19(7):e1010847. doi: 10.1371/journal.pgen.1010847 (PMC10381071; doi:10.1371/journal.pgen.1010847)
Supplement: S1 Table — (DOCX) [file pgen.1010847.s003.docx]

**Table S1:The sequences of siRNA used in this article.**

| **Names** | **Sequences** |
| --- | --- |
| siNC-F | AAAA |
| siNC-R | UUUU |
| siRELL2#1-F | GCUAUGGACUGCACGAACA |
| siRELL2#1-R | UGUUCGUGCAGUCCAUAGC |
| siRELL2#2-F | GCUGCAUCAUCCAGAAUGA |
| siRELL2#2-R | UCAUUCUGGAUGAUGCAGC |
| siRELL2#3-F | GGCCUCCACUUGUCCGUCA |
| siRELL2#3-R | UGACGGACAAGUGGAGGCC |
| siDHX38#1-F | CCCACGACAUCGACCUAAA |
| siDHX38#1-R | UUUAGGUCGAUGUCGUGGG |
| siDHX38#2-F | GGCUAUGACGAGUUCCACA |
| siDHX38#2-R | UGUGGAACUCGUCAUAGCC |
| siDHX38#3-F | GCUCAGCGGAGACAGAUCA |
| siDHX38#3-R | UGAUCUGUCUCCGCUGAGC |
| siRELL2intron4#1-F | GUGUCUCUACCACAGGGA |
| siRELL2intron4#1-R | UCCCUGUGGUAGAGACAC |
| siRELL2intron4#2-F | CUUCAUUCUGGAUGAUGCAG |
| siRELL2intron4#2-R | CUGCAUCAUCCAGAAUGAAG |
