# Supplementary material for: DHX38 restricts chemoresistance by regulating the alternative pre-mRNA splicing of RELL2 in pancreatic ductal adenocarcinoma
Source: PLoS Genet. 2023 Jul 28;19(7):e1010847. doi: 10.1371/journal.pgen.1010847 (PMC10381071; doi:10.1371/journal.pgen.1010847)
Supplement: S2 Table — (DOCX) [file pgen.1010847.s004.docx]

| **Names** | **Sequences** |
| --- | --- |
| RELL2-F | CGGGTGACACACATTGAG |
| RELL2-R | CCTGAGTCCTCCATTCTGT |
| RELL2intron4-F | GTGTCTCTACCACAGGGA |
| RELL2intron4-R | CTTCATTCTGGATGATGCAG |
| DHX38-F | GATTGTGGAGCATCTGGAGGAACTG |
| DHX38-R | GAACGCCATCTGGAGCCTTCTG |
| SF3B4-F | AAATTATGCGGGACCCTGACACAG |
| SF3B4-R | ACGGTGATAGGACGGTTACAGAGG |
| PRPF6-F | GAAGATCATCGACCGAGCCATCAC |
| PRPF6-R | TCCCAGCCCTGTCACATTCCTC |
| HNRNPD-F | GATTGACGCCAGTAAGAACGAGGAG |
| HNRNPD-R | CATTTTCCATTCTTCCCGCTGTGC |
| SNRPB2-F | TTGAAAATGATGGGCAGGCTGGAG |
| SNRPB2-R | GATCTTCATAGCATGGGACGGTGTG |
| ACTB-F | TGGCACCCAGCACAATGAA |
| ACTB-R | CTAAGTCATAGTCCGCCTAGAAGCA |

**Table S2:The sequences of primers used in this article**
