# Supplementary material for: DHX38 restricts chemoresistance by regulating the alternative pre-mRNA splicing of RELL2 in pancreatic ductal adenocarcinoma
Source: PLoS Genet. 2023 Jul 28;19(7):e1010847. doi: 10.1371/journal.pgen.1010847 (PMC10381071; doi:10.1371/journal.pgen.1010847)
Supplement: S3 Table — (DOCX) [file pgen.1010847.s005.docx]

**Table S3:** Data of antibodies used in this article

| **Antibody** | **WB** | **IHC** | **ChIP** | **Specificity** | **Company** |
| --- | --- | --- | --- | --- | --- |
| RELL2 | 1:1000 | 1:200 | - | Rabbit polyclonal | Invitrogen  (PA5-23910) |
| DHX38 | 1:1000 | 1:150 | 1:50 | Rabbit polyclonal | Proteintech  (10098-2-AP) |
| β-actin | 1:2000 | - | - | Rabbit monoclonal | Abcam  (ab8227) |
